# Augmentation genioplasty using discarded bone fragments following proximal segment osteotomy of the ramus in intraoral vertical ramus osteotomy (IVRO)

**DOI:** 10.1186/s40902-024-00433-w

**Published:** 2024-07-19

**Authors:** Sang-Hoon Kang, Chan-Young Lee, Taek-Geun Jun, Min-Jun Kang

**Affiliations:** 1 Department of Oral and Maxillofacial Surgery, National Health Insurance Service Ilsan Hospital, 100 Ilsan-Ro, Ilsan-Donggu, Goyang, Gyeonggi-Do 10444 Republic of Korea; 2 Department of Oral and Maxillofacial Surgery, Gangnam Severance Hospital, Yonsei University College of Dentistry, 211 Eonju-Ro, Gangnam-Gu, Seoul, 06273 Korea

**Keywords:** 3D orthognathic surgery, Augmentation genioplasty, Vertical ramus osteotomy

## Abstract

**Background:**

Based on a three-dimensional (3D) orthognathic simulation, this technical report introduces a method for augmentation genioplasty using a proximal bone fragment of the mandible, which is typically discarded in intraoral vertical ramus osteotomy (IVRO).

**Results:**

A 43-year-old female patient diagnosed with Class III malocclusion, presenting with a protruding mandible and long facial height, underwent surgical treatment. The surgical plan involved mandibular setback position using IVRO and augmentation genioplasty. The 3D orthognathic surgery including augmentation genioplasty simulation was performed. An excessively elongated proximal segment was sectioned following IVRO. The inferior part of the sectioned proximal bone fragment of the mandible was positioned to align with the requirements of advancement genioplasty. After ensuring that the placement of the fragment matched that of the simulated surgery, each bone fragment was fixed. At 1.5 years post-surgery, the grafted bone on the augmentation genioplasty was well maintained, with slight bone resorption.

**Conclusions:**

Augmentation genioplasty using the proximal bone fragment of the mandible, which is typically discarded in IVRO, reduces the surgical complications associated with chin osteotomy. When a secondary genioplasty is required, genioplasty with osteotomy, movement of the cut bone fragments, partial bone-shaving osteotomy, and additional bone grafting are viable options.

## Background

When performing intraoral vertical ramus osteotomy (IVRO) to move the mandible backward the proximal bone segment from the mandible can be harvested and used for grafting during genioplasty [1]. The current trend in orthognathic surgery involves utilizing patient-specific three-dimensional (3D) images from computed tomography (CT) scans for simulation, thereby contributing to the creation of a surgical plan before the actual procedure [2]. Genioplasty is a key component of orthognathic surgery, and an accurate preoperative diagnosis and surgical plan based on simulation are crucial for achieving aesthetic and functional results [3, 4].

In genioplasty, besides directly cutting the mentum and repositioning the bone fragments, bone graft materials or grafts can be applied to the genioplasty area. While various surgical methods exist for augmentation genioplasty, including the use of alloplastic implants or allogenic and xenogenic bone grafts, the importance of considering autogenous bone, which also requires consideration of the donor site, is paramount [5–7].

In cases where the mandible is repositioned posteriorly using IVRO, the lower part of the proximal segment of the ramus is typically removed and discarded [8, 9]. However, during IVRO to move the mandible backward, the proximal bone segment from the mandible can be harvested and used for grafting during genioplasty [1]. This technical report introduces a method for augmentation genioplasty based on a three-dimensional (3D) orthognathic simulation, using a proximal bone fragment of the mandible, which is typically discarded in IVRO.

## Methods

A 43-year-old female patient diagnosed with Class III malocclusion was referred to the Oral and Maxillofacial Surgery Department for Orthognathic Surgery. Cephalometric analysis indicated a protruding mandible and elongated facial height. To address these issues, a treatment plan was devised to rotate the maxilla clockwise and move it in a posterior-superior direction, while also adjusting the setback position of the mandible using IVRO and planning for augmentation genioplasty.

### Simulation of orthognathic surgery including genioplasty using the discarded proximal bone fragment *of ramus* after vertical ramus osteotomy

Facial computed tomography (CT) data were used, which required slices no thicker than 1 mm. Digital Imaging and Communications in Medicine (DICOM) files from the CT images were imported into Mimics software (version 14.0, Materialize, Leuven, Belgium) for 3D reconstruction. The cranio-maxillofacial area of the patient was reconstructed from CT DICOM and Communications in Medicine data, forming a model of the facial skeleton. A final surgical treatment plan was established following virtual surgeries of the maxilla and mandible, ensuring that the positions and shapes of the facial bones were suitable for cephalometric analysis.

**Fig. 1:**
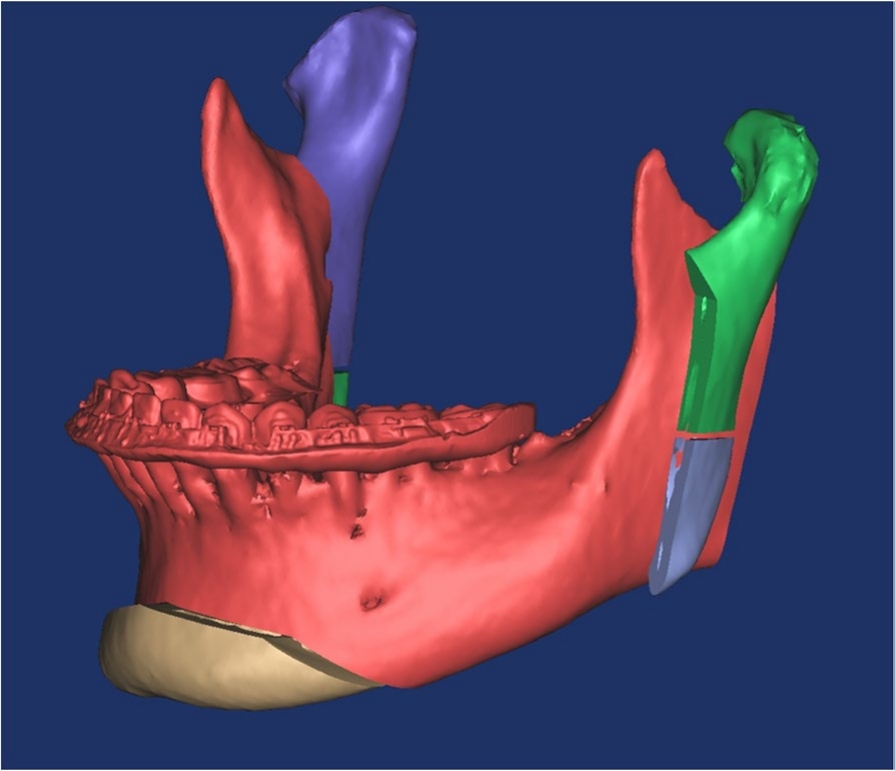
Simulation surgery in which the mandible was positioned posteriorly and advancement genioplasty was performed after vertical ramus osteotomy (VRO). After VRO, the lower part of the proximal segment of the mandible (green on the left, purple on the right) was osteotomized. (Right lower proximal segment inferior part-green, left lower proximal segment inferior part-purple)

The 3D orthognathic surgery with genioplasty simulation was performed considering the planned procedure by the surgeon. Using 3D cephalometric analysis, the maxilla was intended to rotate clockwise and move in a posterior-superior direction, while adjusting the setback position of the mandible using IVRO and planning for augmentation genioplasty. The positions of the mental nerve and corresponding foramen, as well as the distance from the apices of the mandibular anterior teeth, were considered to ensure alignment of the surgical plan with the requirements of maxillary and mandibular orthognathic surgery (Fig. 1). Chin osteotomy was performed to ensure compatibility with the overall orthognathic surgical strategy.

**Fig. 2:**
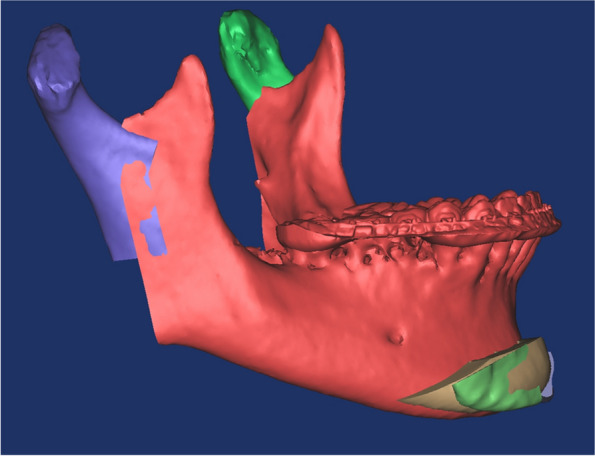
Simulation surgery in which the proximal segment inferior part bone is placed in the area where the advanced genioplasty surgery was performed so that it is similar in size and shape to the advanced genioplasty bone segment

**Fig. 3:**
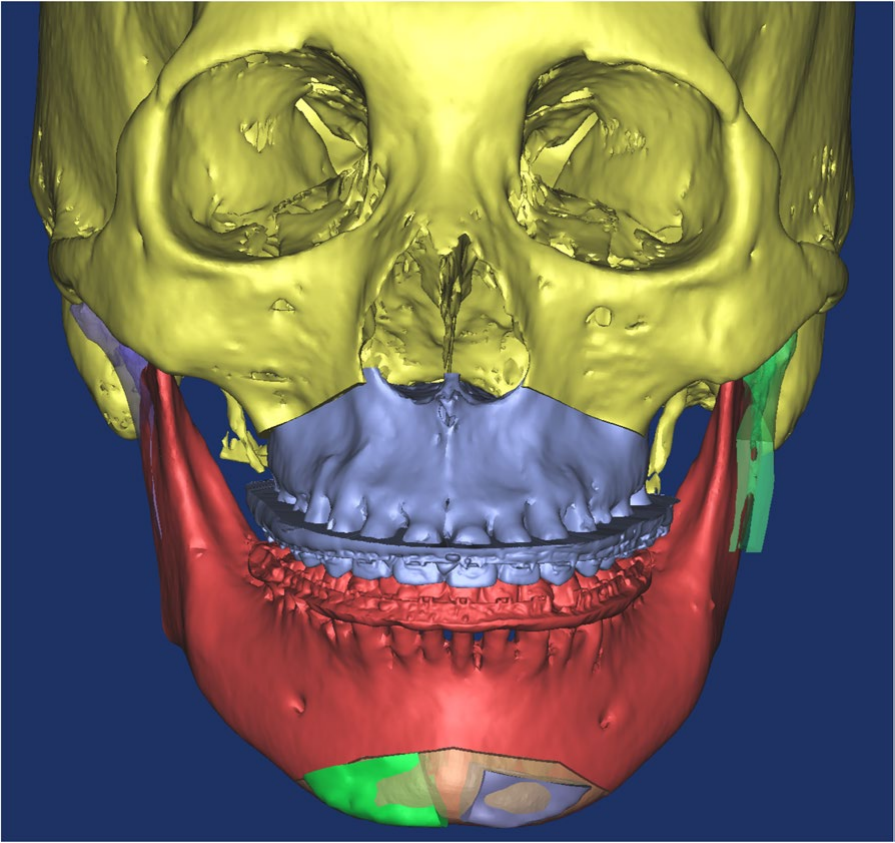
Frontal view after orthognathic surgery simulation. After Le Fort I osteotomy of the maxilla (light purple) and Vertical ramus osteotomy (VRO) of the mandible, the proximal segment inferior part of the mandible was placed in the mentum area and the simulation surgery was performed as planned. (Right lower proximal segment inferior part-green, left lower proximal segment inferior part-purple)

**Fig. 4:**
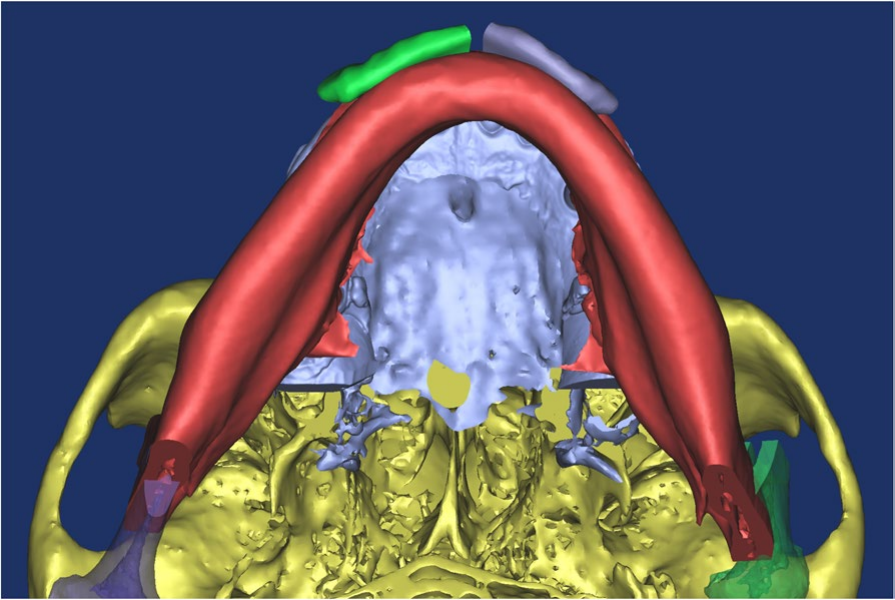
Submentovertex view after orthognathic simulation surgery. Following vertical ramus osteotomy, the osteotomized proximal segment inferior part of the bone was in bone contact with the mentum area, and the planned augmented genioplasty was carried out as planned. (Right lower proximal segment inferior part-green, left lower proximal segment inferior part-purple)

An excessively elongated proximal segment was sectioned following IVRO. The height of the osteotomy can be set at the level of the mandibular border or higher up to the level of the occlusal plane (Fig. 2). In our case, osteotomy was performed at the level of the occlusal plane. The inferior part of the sectioned proximal bone fragment of the mandible was positioned to align with the requirements of advancement genioplasty. The anterior displacement and height of the mandible closely matched (Fig. 3). Considering the convex exterior and concave interior surfaces of the osteotomized inferior part of the sectioned proximal bone fragment, the contact area between the mentum and the sectioned bone was maximized (Fig. 4). In this particular patient, the left medial bone fragment of the mandible was positioned on the left side of the chin, with the right medial bone fragment positioned on the right side. The 3D orthognathic surgery, including augmentation genioplasty simulation, was performed.

### Augmentation genioplasty using the discarded proximal bone fragment of the ramus

**Fig. 5:**
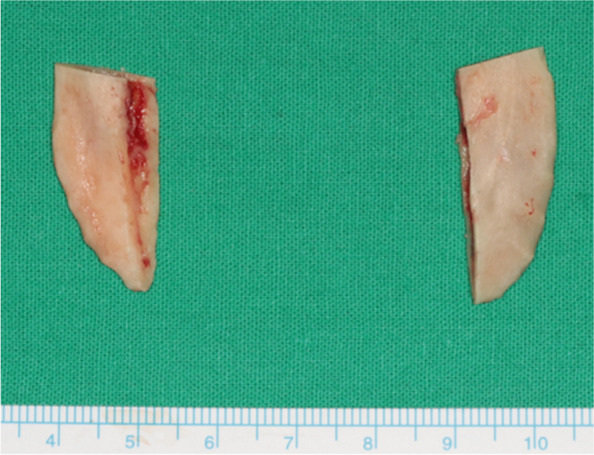
Proximal segment inferior part bone obtained after vertical ramus osteotomy during surgery. Intraoperative photography showing a lateral view of the proximal segment inferior part bone cut to match the occlusal plane level after vertical ramus osteotomy during orthognathic surgery

**Fig. 6:**
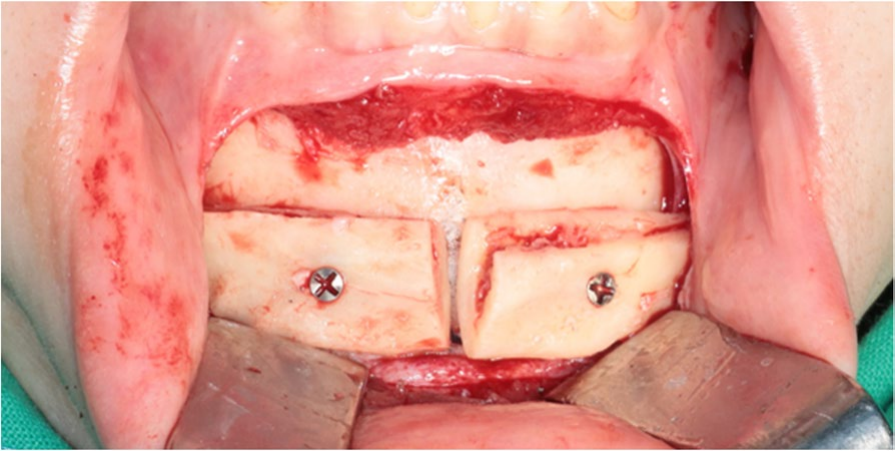
Intraoperative photography showing the proximal segment inferior part bone fragments obtained after vertical ramus osteotomy was fixed with a screw in the mandibular mentum area

After IVRO, the excessively elongated proximal segment was sectioned using a surgical saw while holding the bone with Kocher forceps. In the actual IVRO procedure, which involved the posterior-upward setback movement of the mandible, the inferior part of the proximal segment of the ramus was osteotomized at a height matching the occlusal plane, as established in the simulation surgery (Fig. 5). The chin was exposed, and the obtained bone fragments were placed in the exposed area of the mandible as determined in the simulation surgery, ensuring proper resting of the fragment on the mandible without movement. After ensuring that the placement of the fragment matched that of the simulated surgery, the location of the bone fragment was marked to monitor the movement of the device during surgery. Then, each bone fragment was fixed using mini-screws that were 4–6 mm longer than the graft thickness (Fig. 6). Firm fixation of bone fragments in the desired position was confirmed, followed by suturing. The condition of the soft tissue in the chin area was examined to confirm the extent of advancement.

**Fig. 7:**
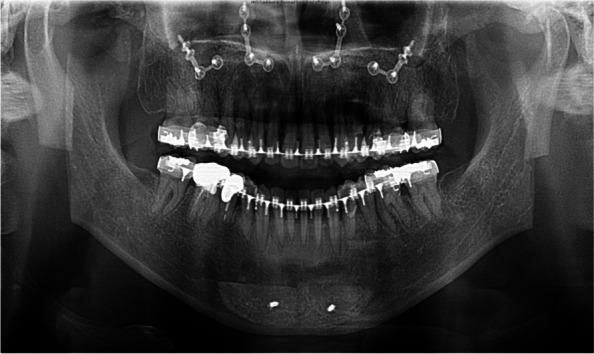
Panoramic radiography immediately after orthognathic surgery shows the inferior part of the proximal segment bones obtained after vertical ramus osteotomy being fixed onto the mentum of the mandible

**Fig. 8:**
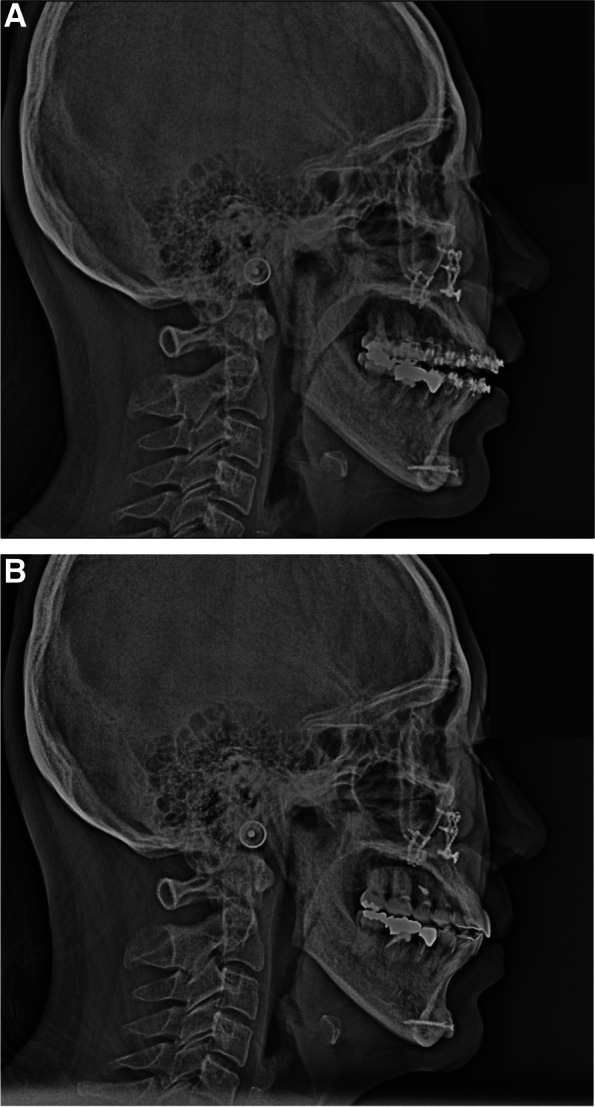
Postoperative cephalometric radiography. **A** Lateral cephalometric radiography immediately after orthognathic surgery. Maxilla was done by Le Fort I osteotomy and positioned postero-superiorly. The mandible was repositioned posteriorly through vertical ramus osteotomy. The inferior part of the proximal segment of the mandible was fixed to the mentum, and the surgery was carried out as planned. **B** Lateral cephalometric radiograph 1.5 years after surgery, grafted proximal segment inferior part bone of the mandible was slightly absorbed and bone fused to the mentum of the mandible

In the panoramic image taken immediately after surgery, the cut area of the proximal bone of the mandibular ramus and the augmented mentum with bone grafting were confirmed (Fig. 7). At 1.5 years post-surgery, the grafted bone on the augmentation genioplasty was well maintained compared to the lateral cephalometric image taken immediately after surgery, although there was slight bone resorption (Fig. 8). The soft tissues in the chin area were maintained according to the planned extent of advancement.

## Discussion

The method described in this case involved transplanting bone fragments from the proximal segment created after IVRO to the genioplasty site during augmentation genioplasty in conjunction with orthognathic surgery. The use of 3D skeletal analysis of facial structures and digital preoperative orthognathic simulation surgery is becoming standardized in orthognathic surgery [2]. To achieve aesthetic and functional results in genioplasty accompanying maxillary and mandibular orthognathic surgery, it is essential to establish a surgical plan through proper diagnosis and simulation before the actual procedure [3, 4].

Augmentation genioplasty using transplantation techniques allows for a variety of grafting materials, making the procedure simpler, with autologous bone being significantly advantageous due to its biocompatibility [1, 6, 10]. However, obtaining autologous bone typically requires separate surgeries, which is a significant drawback. In autologous bone transplantation, consideration must be given to an appropriate donor site from which sufficient bone fragments can be obtained, and the properties of the bone fragments must also be considered. In case of surgery to obtain bone fragments on the donor site, there are preparations in the operating room, surgery time, and complications at the surgical donor site.

This disadvantage can be overcome by utilizing the mandibular bone discarded from IVRO, as introduced in this study. Preoperative simulation surgery allows for the assessment and adjustment of bone fragments to achieve the desired outcome in genioplasty. If the thickness of the fragment is sufficient, the thicker part can be used in augmentation genioplasty according to the planned thickness, considering the amount of marrow and thickness of the cortical bone of the inferior part of the proximal mandibular ramus.

The advantage of the method introduced in this study is that it does not require bone cutting in the chin, thereby reducing surgery time and eliminating bleeding as well as the risk of airway blockage due to swelling or severe bleeding on the lingual side of the chin [11]. Additionally, the risk of root canal treatment due to nerve damage to the anterior teeth from chin bone cutting is not a concern, nor is there nerve damage to the chin [12]. Notably, there are no aesthetic issues with concavities appearing in the forward-moving part of the cutting area of the chin [13]. When autologous bone, xenogeneic bone, or graft material is inserted into the chin area, there is no movement of the muscles attached to the inferior part of the genial tubercle. When genioplasty is performed on patients with sleep apnea, the purpose is to change the muscles genioglossus and geniohyoid attached to the hyoid bone to induce expansion of the hyoid bone and airway and to obtain aesthetic effects. This method is available only in the case of IVRO performing genioplasty using bone fragments discarded from IVRO [14].

In this method, considering the curvature of the cortical bone, fragments from the same area are fixed to the corresponding area on the chin, allowing directional changes or flipping to suit the purpose of genioplasty. Forming a wide bone contact area is advantageous for bone healing; however, even if only some contact is achieved, the remaining area can be filled using particulate bone materials such as commercialized xenogenic bone or other types of grafting material. In this case, bone from the same side was used and fitted well without the need for bone removal owing to the curved outer surface.

Fixation is accomplished using mini-screws; however, considering the possibility of screw breakage, the use of 2.0 mm screws can be considered. Screws longer than the thickness of the grafted bone are employed, utilizing only the outer cortical bone of the chin; long screws that penetrate as far as the lingual side can also be utilized. After positioning the bone fragment as planned in the simulation surgery and marking the location with a pencil, placement was confirmed following fixation per the simulation.

Regarding the efficacy of 3D simulation and guides, using a surgical guide after computer-assisted diagnosis and surgical planning can make the surgery easier, faster, and more accurate in the genioplasty [4]. In genioplasty using osteotomy, depending on the surgical method, inaccuracies in genioplasty can increase due to fractures or insufficient bone removal in the lingual cortex area [15]. Even when using surgical guides, deviations from the desired cutting line can occur due to the oscillation of the cutting tool or its thickness and angle. Although surgical guides can also be used in chin osteotomies, they were not required in the method described in this case.

In genioplasty, the results may differ from expectations due to long-term responses [16, 17]. In this case, resorption of the transplanted bone fragment was observed, but there were no aesthetic problems with the soft tissue; therefore, additional surgery was not planned. When the mandible is positioned posteriorly using setback surgery in mandibular prognathism, the mandible moves posteriorly after IVRO and forward after sagittal split ramus osteotomy [18]. If the mandible is moved posteriorly after IVRO, the position of this part will be different from what was planned before surgery.

However, in cases where additional surgery is required after augmentation genioplasty using graft, there are surgical options available. These include genioplasty with osteotomy and movement of the cut bone fragments, as well as partial bone shaving osteotomy with additional bone grafting. This method is helpful in selecting the surgical method for the secondary surgery when planning or performing secondary genioplasty since no osteotomy was performed and no autologous bone donor surgery was performed.

When performing segment augmentation using horizontal osteotomy for the lower border of the mandible, 76% of the initial advancement amount was maintained, and 24% bone resorption was observed [19]. During a study on segment augmentation using bone fragments obtained from the mandible, undesirable bone resorption was observed in 73.9% of cases during segment augmentation using bone fragments obtained through mandibular angle resection [20]. Among the 46 patients, 5 (10.8%) experienced complete bone loss, 29 (63.1%) had more than 50% bone loss, 10 (21.7%) had less than 50% bone loss, and 2 (4.3%) had mild or no bone loss [20]. As in this case, when vertical height augmentation genioplasty was performed using bone fragments obtained during IVRO, there was a report indicating that the height increased by 4.3 mm, but 0.39 mm was absorbed [1].

Differences between the simulation surgery and actual osteotomy on the ramus during IVRO can occur. Even if osteotomy is performed as planned in the simulation surgery, the amount of bone determined in the simulation may be less than what is needed for genioplasty. In particular, because the thickness of the mandible is limited, slight compensation with allogeneic or xenogeneic bone may be possible if the required bone volume for augmentation genioplasty is large. In augmentation genioplasty cases such as ours, where bone cutting and advancement are performed, bone grafting is possible if a significant amount of advancement is required.

Preoperative surgical simulation is a common approach that offers several benefits in genioplasty. However, in surgeries where replicating the content of the preoperative simulation is challenging, the use of surgical guides can be helpful. Continuous research on various designs for different forms of genioplasty is expected to reduce complications and improve results after genioplasty. Alongside device fabrication, the application and development of computer-assisted surgery, including navigation and robotic surgery, bone graft techniques, and bone graft materials, are expected to advance and be applied in genioplasty.

## Conclusion

The method described in this case involves transplanting bone fragments from the proximal segment created after IVRO to the genioplasty site during augmentation genioplasty in conjunction with orthognathic surgery to reduce the surgical complications associated with osteotomy of the chin. When a secondary genioplasty is required, genioplasty with osteotomy, movement of the cut bone fragments, partial bone-shaving osteotomy, and additional bone grafting are viable options.

## Data Availability

Not applicable.

## Abbreviations

IVRO: Intraoral vertical ramus osteotomy
3D: Three-dimensional
CT: Computed tomography

## References

[1] Kim JY, Kim JK, Park JH, Jung YS (2020) Vertical height augmentation genioplasty using autogenous bone harvested from proximal segments after vertical ramus osteotomy. Br J Oral Maxillofac Surg 58:e124–e126 32636087 10.1016/j.bjoms.2020.06.026

[2] Barretto MDA, Melhem-Elias F, Deboni MCZ (2022) The untold history of planning in orthognathic surgery: a narrative review from the beginning to virtual surgical simulation. J Stomatol Oral Maxillofac Surg 123:e251–e259 35413462 10.1016/j.jormas.2022.04.002

[3] Jiang Y, Yang B, Yang F, Li B, Ma H, Huang Q et al (2021) One-half wedge osteotomy genioplasty for correction of chin deviation based on three-dimensional computed tomography measurements and simulation. J Craniofac Surg 32:1496–1499 33427787 10.1097/SCS.0000000000007431

[4] Lim SH, Kim MK, Kang SH (2015) Genioplasty using a simple CAD/CAM (computer-aided design and computer-aided manufacturing) surgical guide. Maxillofac Plast Reconstr Surg 37:44 26636050 10.1186/s40902-015-0044-y PMC4656692

[5] Bertossi D, Albanese M, Nocini PF, D’Agostino A, Trevisiol L, Procacci P (2013) Sliding genioplasty using fresh-frozen bone allografts. JAMA Facial Plast Surg 15:51–57 23147279 10.1001/jamafacial.2013.224

[6] Kerbrat A, Ferri J (2021) Augmentation genioplasty using a third molar as a bone graft: an alternative surgical technique. J Craniofac Surg 32:e393–e394 33427774 10.1097/SCS.0000000000007425

[7] Kim GJ, Jung YS, Park HS, Lee EW (2005) Long-term results of vertical height augmentation genioplasty using autogenous iliac bone graft. Oral Surg Oral Med Oral Pathol Oral Radiol Endod 100:e51–e57 16122647 10.1016/j.tripleo.2005.04.020

[8] Kang SH, Kang MJ, Kim MJ, Kim MK (2022) Changes in facial width according to the ostectomy level of the proximal bone segment in intraoral vertical ramus osteotomy for mandibular prognathism. Maxillofac Plast Reconstr Surg 44:16 35435520 10.1186/s40902-022-00347-5 PMC9016097

[9] Jung HD, Kim SY, Park HS, Jung YS (2014) Modification of intraoral vertical ramus osteotomy. Br J Oral Maxillofac Surg 52:866–867 24906250 10.1016/j.bjoms.2014.04.020

[10] Perez Villar A, Krebs Rodrigues FL, Gomes Patrocinio L (2020) Sliding genioplasty using mastoid bone interpositional graft. Facial Plast Surg Aesthet Med 22:483–485 32552039 10.1089/fpsam.2020.0147

[11] Choi BK, Lee SS, Yun IS, Yang EJ (2023) Vascular anatomy for the prevention of sublingual hematomas: life-threatening complication of genioplasty. J Craniofac Surg 34:1308–1311 36730838 10.1097/SCS.0000000000009104

[12] Avelar RL, Sá CD, Esses DF, Becker OE, Soares EC, de Oliveira RB (2014) Unusual complication after genioplasty. J Craniofac Surg 25:e180–e182 24621765 10.1097/SCS.0000000000000618

[13] Oranges CM, Grufman V, di Summa PG, Fritsche E, Kalbermatten DF (2023) Chin augmentation techniques: a systematic review. Plast Reconstr Surg 151:e758–e771 10.1097/PRS.0000000000010079 36729154

[14] Song SA, Chang ET, Certal V, Del Do M, Zaghi S, Liu SY et al (2017) Genial tubercle advancement and genioplasty for obstructive sleep apnea: a systematic review and meta-analysis. Laryngoscope 127(4):984–992 27546467 10.1002/lary.26218

[15] Kang SH, Lee JW, Lim SH, Kim YH, Kim MK (2014) Validation of mandibular genioplasty using a stereolithographic surgical guide: in vitro comparison with a manual measurement method based on preoperative surgical simulation. J Oral Maxillofac Surg 72:2032–2042 24780609 10.1016/j.joms.2014.03.002

[16] Shaughnessy S, Mobarak KA, Høgevold HE, Espeland L (2006) Long-term skeletal and soft-tissue responses after advancement genioplasty. Am J Orthod Dentofacial Orthop 130:8–17 16849066 10.1016/j.ajodo.2004.11.035

[17] George JA, Kannan A, Kailasam V (2022) Long-term hard and soft tissue response following isolated genioplasty: a systematic review. Oral Maxillofac Surg 26:195–203 34383152 10.1007/s10006-021-00991-7

[18] Chen CM, Hwang DS, Hsiao SY, Chen HS, Hsu KJ (2021) Skeletal stability after mandibular setback via sagittal split ramus osteotomy verse intraoral vertical ramus osteotomy: a systematic review. J Clin Med 10(21):4950 34768470 10.3390/jcm10214950 PMC8584578

[19] Polido WD, de Clairefont RL, Bell WH (1991) Bone resorption, stability, and soft-tissue changes following large chin advancements. J Oral Maxillofac Surg 49:251–256 1995814 10.1016/0278-2391(91)90214-7

[20] Tang X, Gui L, Zhang Z (2009) Analysis of chin augmentation with autologous bone grafts harvested from the mandibular angle. Aesthet Surg J 29:2–5 19232998 10.1016/j.asj.2008.11.004

